# Risk factors of poor mid-term shoulder functional outcomes of osteosynthesis using antegrade intramedullary nailing for humeral shaft fractures: a retrospective study with a minimum 5-year follow-up

**DOI:** 10.1186/s12891-024-07572-1

**Published:** 2024-06-08

**Authors:** Ryogo Furuhata, Atsushi Tanji, Soichiro Nakamura

**Affiliations:** Department of Orthopaedic Surgery, Ashikaga Red Cross Hospital, 284-1 Yobe-cho, Ashikaga, 326-0843 Tochigi Japan

**Keywords:** Humeral shaft fracture, Intramedullary nail, Shoulder, Outcome, Older age, Nail protrusion

## Abstract

**Background:**

Osteosynthesis using antegrade intramedullary nailing for humeral shaft fractures yields satisfactory bone union rates; however, it may adversely affect postoperative shoulder function. To date, factors affecting mid- or long-term shoulder functional outcomes following intramedullary nail fixation have not been clarified. In this study, we aimed to identify the risk factors for poor mid-term functional outcomes over 5 years postoperatively following antegrade intramedullary nail osteosynthesis for humeral shaft fractures.

**Methods:**

We retrospectively identified 33 patients who underwent surgery using an antegrade intramedullary nail for acute traumatic humeral shaft fractures and were followed up for at least 5 years postoperatively. We divided the patients into clinical failure and no clinical failure groups using an age- and sex-adjusted Constant score of 55 at the final follow-up as the cutoff value. We compared preoperative, perioperative, and postoperative factors between the two groups.

**Results:**

Five of the 33 patients had poor shoulder functional outcomes (adjusted Constant score < 55) at a mean follow-up of 7.5 years postoperatively. Proximal protrusion of the nail at the time of bone union (*P* = 0.004) and older age (*P* = 0.009) were significantly associated with clinical failure in the univariate analyses. Multivariate analysis showed that proximal protrusion of the nail (*P* = 0.031) was a risk factor for poor outcomes.

**Conclusions:**

The findings of this study provide new information on predictive factors affecting mid-term outcomes following osteosynthesis using antegrade nails. Our results demonstrated that proximal protrusion of the nail was significantly associated with poor mid-term functional shoulder outcomes. Therefore, particularly in older adults, it is essential to place the proximal end of the intramedullary nail below the level of the articular cartilage.

## Background

Osteosynthesis using an intramedullary nail for humeral shaft fractures provides solid stability and good load sharing, while minimizing soft tissue damage and preserving the periosteal blood supply at the fracture site [1, 2], resulting in satisfactory postoperative bone union rates [3–11]. Contrarily, antegrade intramedullary nailing has been reported to cause more shoulder complications, such as shoulder pain and decreased range of shoulder motion or muscle strength, compared with plate fixation [5, 7, 9, 11, 12].

Previous studies have suggested that the risk factors for poor shoulder functional outcomes following osteosynthesis using an antegrade intramedullary nail include proximal protrusion of the nail [4, 13–15], nonunion [14, 16], older age [17], radial nerve injury [4], brachial plexus nerve injury [4], and ipsilateral superior-limb fracture [3]. In addition, the occurrence of nonunion or delayed union is associated with older age [18], time from injury to surgery [19], distraction of fracture [3, 14], and fracture type (transverse fracture) [3]. However, these previous reports on the postoperative outcomes of humeral shaft fractures were based on short-term results in patients with a mean follow-up time of 1–3 years after surgery [3, 4, 13–19]; therefore, the factors affecting mid- or long-term shoulder functional outcomes remain largely unknown.

Therefore, in this study, we aimed to determine the factors that influence the mid-term shoulder functional outcomes of antegrade intramedullary nail osteosynthesis for humeral shaft fractures by investigating the postoperative results in patients with a minimum follow-up duration of 5 years.

## Methods

### Study design and patients

The independent ethics committee of Ashikaga Red Cross Hospital approved the study protocol (No. 2022-33). This retrospective study included adult patients (with closed epiphysis) who underwent osteosynthesis using an antegrade intramedullary nail for acute humeral shaft fractures within 3 weeks of injury at a single general hospital between 2011 and 2018. Patients who could not be evaluated for shoulder functional outcomes at > 5 years postoperatively and those with pathological fracture, open fracture, a history of shoulder surgery, and paralysis of the affected upper extremity due to cerebral infarction or other causes were excluded.

### Surgical procedure

Six orthopedic surgeons performed the surgeries. In all cases, the surgeries were performed in the beach-chair position under general anesthesia. Osteosynthesis was performed using a deltoid split approach. We made a 1–2 cm incision with a scalpel in the direction of the muscle fibers with the supraspinatus tendon, preserving its insertion at the greater tuberosity. We inserted a guide pin from the apex of the humeral head and opened the medullary canal using an awl. An intramedullary nail was inserted under fluoroscopic guidance. The implants used in this study were the MultiLoc humeral nail (DePuy Synthes, Oberdorf, Switzerland), Trigen humeral nails (Smith & Nephew, Watfold, UK), and Polarus2 humeral nail (Acumed, Hillsboro, OR, USA). After manual reduction and nail insertion, distal and proximal locking screws were inserted. The number of screws depended on the surgeon’s judgment, based on the stability of the fixation of the fracture site. The end cap was then screwed. We repaired the supraspinatus tendon with 2–4 single stitches using no. 0 Surgilon™ (Medtronics, Dublin, Ireland).

After surgery, the patients wore a sling for 1–2 weeks, during which passive range-of-motion training was started, while active motion training was started at 4–6 weeks postoperatively.

### Outcome measures

We evaluated postoperative shoulder functional outcomes using the Constant score [20], American Shoulder and Elbow Surgeons (ASES) score [21], and visual analog scale (VAS). One examiner with 10 years of experience in shoulder surgery, who was not involved in the surgery, evaluated the outcomes. Constant scores were adjusted for age and sex [22]. Based on previous reports on surgical outcomes for proximal humeral fractures [23], an adjusted constant score of < 55 was considered a poor outcome and defined as “clinical failure” in this study.

The explanatory variables included preoperative factors (age, sex, affected side of the arm, smoking history, diabetes, body mass index (BMI), time from injury to surgery, time from surgery to final follow-up, preoperative radial nerve injury, position in the shaft, fracture type, Arbeitsgemeinschaft für Osteosynthesefragen (AO) classification, and local osteoporosis), perioperative factors (operative time, blood loss, and nail design), and postoperative factors (fracture gap, delayed union, and proximal protrusion of the nail). A single examiner, blinded to postoperative shoulder functional outcomes results, evaluated these variables based on past clinical notes and plain radiographic images. We measured the average cortical bone thickness at two sites of the humerus, based on a previous report, and defined an average proximal humerus cortical thickness of 6 mm as the potential threshold value for predicting local osteoporosis [24]. Nail design was classified into straight nails inserted through the apex of the humeral head (MultiLoc and Trigen) and a lateral curved nail inserted from 4º lateral to the apex of the humeral head (Polarus2). The fracture gap immediately after surgery was measured on plain radiographs obtained immediately after surgery as the shortest distance between the proximal and distal bone fragments, according to a previous report [25]. Delayed union was defined as bone union occurring after 26 weeks [26]. Proximal nail protrusion was defined as a protrusion of the end of the nail more than 1 mm above the humeral head in either the anteroposterior view or scapular-Y view of the shoulder plain radiograph taken at the bone union.

Patients were divided into a clinical failure group and no clinical failure group, using an adjusted Constant score of 55 at the final follow-up as the cutoff value. We compared the two groups’ average and frequency of the explanatory variables in the univariate analysis. Significant baseline variables in the univariate analyses and reported risk factors for short-term poor outcomes (proximal protrusion of the nail [4, 13–15], delayed union [14, 16], older age [17], and preoperative radial nerve injury [4]) were included in the multivariate models.

### Statistical analysis

All statistical analyses were conducted using SPSS software (version 25.0*, IBM, Armonk, NY, USA). The continuous data are presented as mean ± standard deviation. The categorical data are presented as number and percentage. We used the bootstrap yuen-welch-t-test to compare the averages of continuous values (age, BMI, time from injury to surgery, time from surgery to final follow-up, operative time, blood loss, fracture gap, adjusted Constant score, ASES shoulder score, VAS, and range of motion). We used Fisher’s exact test (sex, affected side of injury, smoking history, diabetes, preoperative radial nerve injury, position in the shaft, nail design, delayed union, and proximal protrusion of the nail) or chi-square test (fracture type and AO classification) to compare the proportions. Multivariate analysis was performed using logistic regression analysis to identify the independent predictors of mid-term poor outcomes. Regression model fit was estimated using the Hosmer–Lemeshow goodness-of-fit test. Statistical significance was set at *P* < 0.05.

## Results

We identified 54 patients who met our inclusion criteria. Of these, 16 patients were excluded due to loss to follow-up (six patients died, five patients relocated, and five patients self-interrupted), two patients due to pathological fracture, one patient due to open fracture, and two patients due to paralysis of the affected upper extremity caused by cerebral infarction. Thus, a total of 33 patients were included in this study. Patient characteristics are shown in Table 1. The mean age at the time of surgery was 59.9 ± 19.4 (range 17–90) years. The mean time from injury to surgery was 3.8 ± 2.9 (range 1–15) days. The fracture type was proximal third in 22 patients and the middle third in 11. None had a distal third fracture. None of the patients underwent nail removal during the 5-year postoperative period.

**Table 1:** Patient Characteristics

	Number of Patients (%) (*N* = 33)
Sex	
Female	22 (67%)
Male	11 (33%)
Affected side of arm	
Dominant arm	16 (48%)
Non-dominant arm	17 (52%)
Smoking	3 (9%)
Diabetes	5 (15%)
Position in shaft	
Proximal	22 (67%)
Middle	11 (33%)
Fracture type	
Oblique	8 (24%)
Spiral	6 (18%)
Transverse	15 (45%)
Segmented	4 (12%)
AO classification	
A	27 (82%)
B	3 (9%)
C	3 (9%)

AO = Arbeitsgemeinschaft für Osteosynthesefragen

**Fig. 1:**
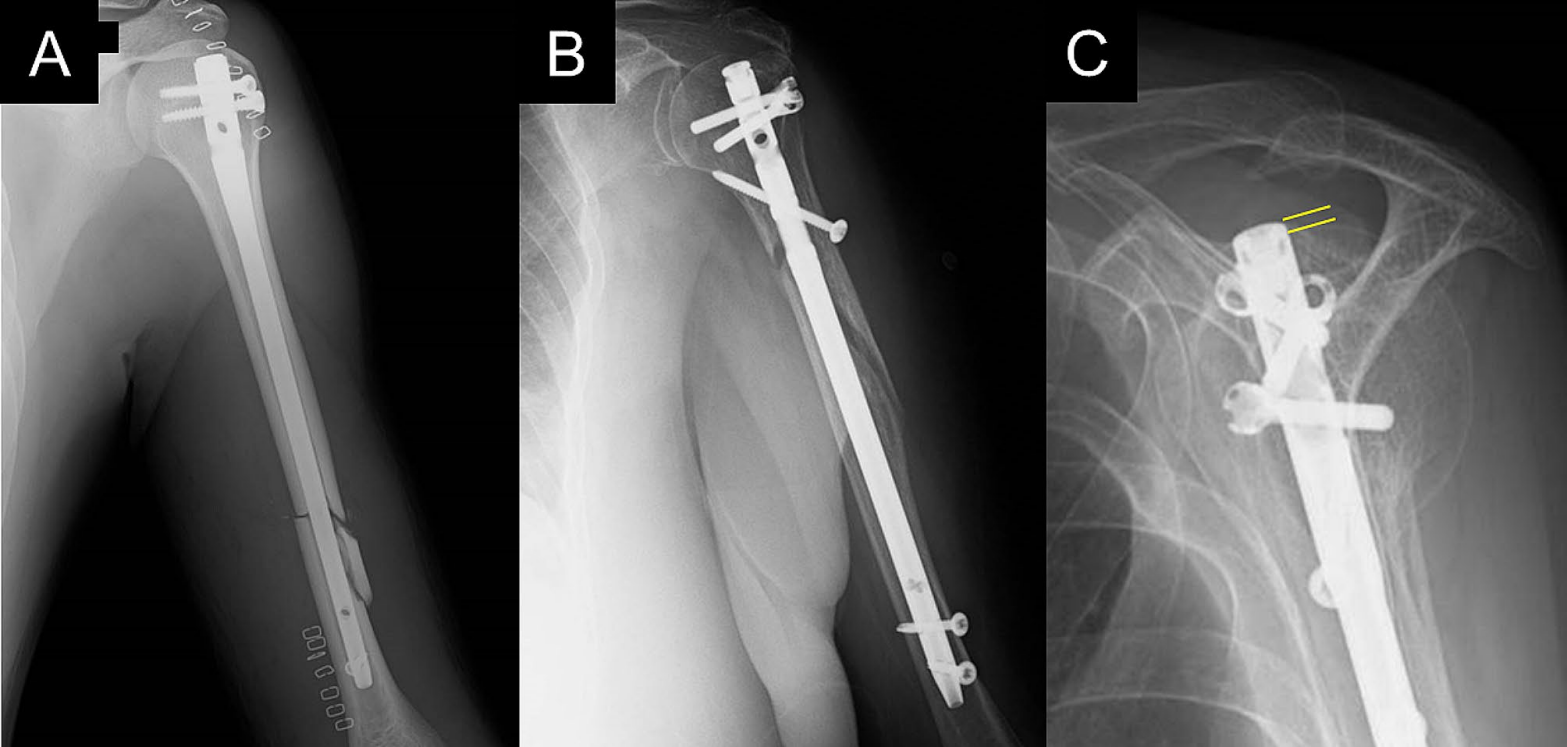
Postoperative radiographs of the representative cases. (**A**) Immediate postoperative radiograph of the patient with preoperative radial nerve palsy. Symptoms of the radial nerve injury improved within 1 year postoperatively, and the Constant score was 100 at 9 years postoperatively. (**B**) Radiograph of the patient presenting with delayed bone union at 26 weeks postoperatively. Subsequently, bone union was observed at 1.5 years postoperatively, and the adjusted Constant score was 95 at 6 years postoperatively. (**C**) Scapula-Y view of the shoulder radiograph of the patient presenting with a nail protrusion of 2 mm above the humeral head at the time of bone union (yellow lines). Six years postoperatively, the patient had residual severe shoulder pain and an adjusted Constant score of 54

The mean follow-up period was 7.5 ± 2.1 (range 5.0–11.4) years. The mean adjusted Constant score, ASES shoulder score, and VAS at the last follow-up were 86.2 ± 15.7 (range 52–100), 84.3 ± 17.1 (range 37–100), and 0.86 ± 1.34 (range 0–6.0) cm, respectively. The mean range of shoulder motion for anterior elevation was 138 ± 24 (range 80–160) ° and external rotation was 46 ± 12 (range 20–70) °. All patients with traumatic radial nerve injury showed improvement over time, with only mild sensory deficits remaining at the final follow-up (Fig. 1A). Iatrogenic nerve injury was not observed postoperatively. Five patients experienced delayed bone union at 26 weeks postoperatively; however, all patients eventually achieved union within two years after surgery (Fig. 1B). Seven patients presented with proximal protrusion of the nail at the bone union in the scapular Y view of the shoulder radiograph (Fig. 1C).

In this study, five patients were classified into the clinical failure group (adjusted Constant score < 55) and 28 patients into the no clinical failure group (adjusted Constant score ≥ 55). The mean ASES shoulder score and range of anterior elevation and external rotation were significantly lower in the clinical failure group than in the no clinical failure group (*P* = 0.001, *P* = 0.001, *P* = 0.014, respectively) (Table 2).

**Table 2:** Comparison of Clinical Outcome Score and Range of Shoulder Motion by Occurrence of Clinical Failure

	Clinical failure (*N* = 5)	No clinical failure (*N* = 28)	*P*-value
Adjusted Constant score	53.8 ± 1.0	92.0 ± 8.5	0.001
ASES shoulder score	49.8 ± 8.0	90.5 ± 9.0	0.001
VAS (cm)	2.50 ± 1.95	0.46 ± 0.91	0.053
Range of shoulder motion			
Anterior elevation (º)	99 ± 14	145 ± 19	0.001
External rotation at sides (º)	35 ± 11	48 ± 12	0.014

Values are presented as means and standard deviations. † ASES = American Shoulder and Elbow Surgeons; VAS = visual analog scale

For the preoperative factors, the mean age at surgery was significantly higher in the clinical failure group than in the no clinical failure group (76.8 ± 11.6 years vs. 56.9 ± 19.0 years, respectively, *P* = 0.009; Table 3). For the perioperative and postoperative factors, the ratio of proximal protrusion of the nail was significantly higher in the clinical failure group than in the no clinical failure group (80% vs. 11%, respectively, *P* = 0.004; Table 4).

**Table 3:** Comparison of Preoperative Factors by Occurrence of Clinical Failure

	Clinical failure (*N* = 5)	No clinical failure (*N* = 28)	*P*-value
Age * (years)	76.8 ± 11.6	56.9 ± 19.0	0.009
Sex †			> 0.999
Female	3	19	
Male	2	9	
Affected side of arm †			0.335
Dominant arm	1	15	
Non-dominant arm	4	13	
Smoking †	0	3	> 0.999
Diabetes †	0	5	0.569
BMI * (kg/m^2^)	22.9 ± 5.9	23.5 ± 5.6	0.841
Time from injury to surgery * (days)	2.4 ± 1.5	4.1 ± 3.0	0.146
Time from surgery to final follow up * (years)	6.0 ± 1.1	7.7 ± 2.1	0.055
Preoperative radial nerve injury †	0	2	> 0.999
Position in shaft †			0.144
Proximal	5	17	
Middle	0	11	
Fracture type †			0.529
Oblique or spiral	1	13	
Transverse	3	12	
Segmented	1	3	
AO classification †			0.520
A	4	23	
B	0	3	
C	1	2	
Local osteoporosis †	4	12	0.175

* Values are presented as means and standard deviations. † Values are presented as the number of patients. BMI = body mass index, AO = Arbeitsgemeinschaft für Osteosynthesefragen

**Table 4:** Comparison of Perioperative and Postoperative Factors by Occurrence of Clinical Failure

	Clinical failure (*N* = 5)	No clinical failure (*N* = 28)	*P*-value
Operative time * (minutes)	93.2 ± 17.3	93.0 ± 42.7	0.938
Blood loss * (g)	30.0 ± 26.9	86.0 ± 108.2	0.070
Nail design †			0.302
Straight	5	20	
Lateral curved	0	8	
Fracture gap immediately after surgery * (mm)	0.9 ± 0.9	1.6 ± 2.2	0.431
Delayed union at 26 weeks after surgery †	2	3	0.155
Proximal protrusion of the nail at the time of union †	4	3	0.004

* Values are presented as means and standard deviations. † Values are presented as the number of patients

Multivariate analysis showed that proximal protrusion of the nail (odds ratio [OR], 121.8; 95% confidence interval [CI], 1.5–9657; *P* = 0.031) was a risk factor for poor mid-term shoulder functional outcomes (Table 5). The Hosmer-Lemeshow goodness-of-fit test showed no significant difference from the good model fit (*P* = 0.661).

**Table 5:** Multivariate Predictors of Poor Mid-term Shoulder Functional Outcomes

Variables	Multivariate Predictors	
	Odds Ratio (95% CI)	*P*-value
Proximal protrusion of the nail at the time of union	121.8 (1.5–9657)	0.031
Age	1.2 (0.97–1.5)	0.100
Delayed union at 26 weeks after surgery	0.98 (0.002-551)	0.995
Preoperative radial nerve injury	< 0.001	> 0.99

CI, confidence interval

## Discussion

In this study, we investigated the postoperative shoulder functional outcomes of patients who underwent osteosynthesis using an intramedullary nail for humeral shaft fractures with a minimum follow-up duration of five years. Subsequently, we identified proximal protrusion of the intramedullary nail and older age as risk factors for poor mid-term outcomes in the univariate analyses. Multivariate analysis showed that proximal protrusion of the nail was a risk factor for poor outcomes.

We found that patients with proximal protrusion of the nail on plain radiography at the time of bone union had significantly lower shoulder functional scores. Several previous studies have suggested an association between proximal nail protrusion and clinical failure [4, 13–15]; however, this is the first study to show a significant association. Proximal nail protrusion is thought to cause impingement of the nail on the rotator cuff or subacromial space, leading to persistent shoulder pain and loss of range of motion [4, 13–15, 27]. Although placing the proximal end of the nail to anchor in the zone of dense subchondral bone is of critical importance in countering varus displacing force [28, 29], this study suggests that the nail should be inserted into the humeral head to avoid protrusion above the level of the articular cartilage. In cases where proximal nail protrusion remains at the time of bone union, nail removal has been reported to improve symptoms [7, 18] and can be a treatment option in such cases.

This study also showed that older age was significantly associated with poor mid-term shoulder functional outcomes in the univariate analysis. A previous study reported that all patients with unsatisfactory shoulder functional outcomes within two years after intramedullary nail fixation were older than 78 years [17], which concurs with the results of the present study. In addition, another study, including patients who underwent conservative therapy, plate fixation, or intramedullary nailing demonstrated an association between older age and poor shoulder function at 26 weeks and 52 weeks following injury [30]. However, multivariate analysis showed no significant association between older age and poor outcomes, possibly because of the confounding effect between nail protrusion and older age due to the effect of rotator cuff degeneration on the healing of the rotator cuff damaged during nail insertion and the osteoporotic loss of cortical bone in older age [18].

Unlike in a previous report [4], preoperative radial nerve injury was not significantly associated with clinical failure in this study. This disparity was attributed to the differences in the postoperative follow-up time. In this study, all patients with traumatic radial nerve injury improved over time, with only mild sensory deficits remaining at more than five years postoperatively, suggesting that preoperative radial nerve injury may affect the short-term shoulder functional outcomes and not the mid-term outcomes.

In addition, our results are different from those of previous reports showing an association between nonunion at six months after surgery and poor postoperative functional outcomes [13, 16]. The differences in the postoperative follow-up time can partly explain this discrepancy. All patients in this study who did not achieve union six months after surgery eventually achieved union within two years after surgery. This raises the possibility that the final bone union had little effect on the mid-term outcomes.

The strength of this study is that it evaluated mid-term shoulder functional outcomes following osteosynthesis for humeral shaft fractures. Previous studies on the postoperative outcomes of humeral shaft fractures have evaluated shoulder function at 1–3 years postoperatively [1, 2, 12]; till date, no study has assessed shoulder functional outcomes at more than five years postoperatively.

However, this study had some limitations. First, the cohort of patients available for analysis was small; therefore, our results may have included the effect of β-error. However, the sample size of most reported studies has been 20–40 patients [1, 2, 12], and our sample size is comparable to these studies. Second, due to the study’s observational nature, biases from unobserved differences may have affected the outcomes. For instance, although six surgeons performed the operations in this study, their skill levels were not taken into consideration. Moreover, the fact that the choice of implants depends on the surgeon’s preference could be a limitation of this study. Third, 16 patients were excluded owing to loss to follow-up, which may decrease the generalizability of the study results.

## Conclusion

This study provides new information on predictive factors affecting mid-term outcomes following osteosynthesis using an antegrade nail. Our findings demonstrated that proximal protrusion of the nail was significantly associated with poor mid-term shoulder functional outcomes. Therefore, particularly in older adults, it is essential to place the proximal end of the intramedullary nail below the level of the articular cartilage.

## Data Availability

Data supporting this study’s findings are available from the corresponding author on reasonable request.

## Abbreviations

ASES: American Shoulder and Elbow Surgeons
VAS: Visual analog scale
BMI: Body mass index
AO: Arbeitsgemeinschaft für Osteosynthesefragen
OR: Odds ratio
CI: Confidence interval
